# Preparedness of Frontline Doctors in Jordan Healthcare Facilities to COVID-19 Outbreak

**DOI:** 10.3390/ijerph17093181

**Published:** 2020-05-02

**Authors:** Aiman Suleiman, Isam Bsisu, Hasan Guzu, Abeer Santarisi, Murad Alsatari, Ala’ Abbad, Ahmad Jaber, Taima’a Harb, Ahmad Abuhejleh, Nisreen Nadi, Abdelkarim Aloweidi, Mahmoud Almustafa

**Affiliations:** 1 Department of Anesthesia and Intensive Care, School of Medicine, The University of Jordan, Amman 11942, Jordan; Isam_Bsisu@hotmail.com (I.B.); Hasangu91@hotmail.com (H.G.); muradalsatari@gmail.com (M.A.); alaaabbad91@gmail.com (A.A.); dr.ahmed.jaber@gmail.com (A.J.); taim.harb@gmail.com (T.H.); Ahmadabuhijleh55@gmail.com (A.A.); Nisreen1991@ymail.com (N.N.); akaloweidi@hotmail.com (A.A.); m.al-mustafa@ju.edu.jo (M.A.); 2 Department of Emergency Medicine and Accidents, School of Medicine, The University of Jordan, Amman 11942, Jordan; abeer_santarisi@yahoo.com

**Keywords:** pandemic preparedness, awareness, frontline doctors, COVID-19

## Abstract

The number of COVID-19 (Coronavirus Disease of 2019) cases in Jordan is rising rapidly. A serious threat to the healthcare system appears on the horizon. Our study aims to evaluate preparedness of Jordanian frontline doctors to the worsening scenario. It has a questionnaire-based cross-sectional structure. The questionnaire was designed to evaluate preparedness according to knowledge about virus transmission and protective measures, adherence to protection guidelines, and psychological impacts affecting doctors. Institutional factors affecting doctors’ readiness like adopting approach protocols and making protection equipment available were investigated; 308 doctors from different healthcare facilities participated (response rate: 53.9%). Approximately 25% of doctors (n = 77) previously took care of COVID-19 patients, and 173 (56.2%) have institutional COVID-19 approach protocols. Only 57 doctors (18.5%) reported all PPE (Personal Protective Equipment) available. The self-reported score of preparedness to deal with COVID-19 patients was 4.9 ± 2.4. Doctors having institutional protocols for dealing with COVID-19 cases and those with sustained availability of PPE reported higher scores of preparedness (5.5 ± 2.3 and 6.2 ± 2.1 with *p* < 0.001, respectively). Correlations with knowledge score, adherence to PPE score, and psychological impacts were investigated. The study revealed multiple challenges and insufficiencies that can affect frontline doctors’ preparedness. Policy makers are urged to take these findings into consideration and to act promptly.

## 1. Introduction

COVID-19 (Coronavirus Disease of 2019) is a new type of virus that has the potential to cause severe respiratory disease [1]. The first encounter of the disease was in the city of Wuhan, China, in December 2019, after which a pandemic emerged and spread all over the world [1]. On 12 March 2020, the World Health Organization (WHO) announced that COVID-19 is categorized as a pandemic [2]. As of 30 March 2020, more than 750,000 positive cases were identified across 170 countries with more than 36,000 reported deaths [3]. The virus may have originated in bats [4]. In most severe cases, patients can develop pneumonia that progresses to acute respiratory distress syndrome (ARDS) requiring mechanical ventilation [5]. 

In Jordan, the first case of COVID-19 was identified on 2 March 2020 in a traveler who had returned from Italy two weeks before quarantine procedures [6]. As of March 23, the starting date of our study, officials announced that the number of cases had reached 127 and that the country had yet to move into the acceleration phase of the epidemic curve. Jordan is a middle-income country with a population exceeding 10 million [7]. Advanced medical care is provided in over 106 tertiary hospitals distributed across the country with about 12,081 beds capacity (1.8 beds per 1000 people) [8]. Jordan is considered a leading country in healthcare services in the Middle East with many global ranks and awards [9,10]. Since March 16, one of the world’s strictest lockdowns took place in the country and five tertiary hospitals were designated to provide medical care for suspected/diagnosed COVID-19 patients [11].

Since the number of COVID-19 cases in Jordan is rising rapidly, a serious threat to the healthcare system appears on the horizon. Adding to availability of equipment, the preparedness of frontline doctors to the impact of the outbreak is what guarantees the system to function properly and efficiently. Our study aims to evaluate the awareness and readiness of these doctors to the worsening scenario in Jordan, a limited-resources country. To the best of our knowledge, this is the first study to evaluate the preparedness of frontline doctors to COVID-19 outbreak in Jordan and in the Middle East.

## 2. Materials and Methods 

### 2.1. Study Design

This is a questionnaire-based cross-sectional study. Our target sample was Jordanian doctors who might be in first contact with COVID-19-positive patients. We identified 571 doctors that were assigned to missions that deal directly with COVID-19 patients. The sample included general practitioners, resident doctors, and specialists. The specialties allocated to first contact with COVID-19 patients were emergency medicine and accidents, anesthesia and intensive care, internal medicine, ENT (ear, nose, and throat), and family medicine. All healthcare sectors which were or might be involved in taking care of COVID-19 patients were involved; these included university hospitals, governmental hospitals, military hospitals, and private hospitals. 

### 2.2. Questionnaire

Our questionnaire was designed to evaluate awareness and readiness of frontline doctors to deal with COVID-19 patients. It was web-based and filled using Google forms We collected data regarding three main aspects of preparedness. Firstly, knowledge and awareness of transmission routes, protection guidelines, and emergency approaches were assessed using five questions, with four points per question. The overall score was then converted to a ten-point score. Secondly, adherence to the PPE (Personal Protective Equipment) guidelines by the CDC (Centers for Disease Control and Prevention) [12] was evaluated using three questions, with an overall score of 12 that was then converted to a ten-point score. In addition, we studied the availability of PPE and the application of institutional protocols for dealing with COVID-19-positive or suspected patients. We also investigated psychological impacts and interactions affecting the preparedness of involved doctors. Moreover, a self-reported 11-point score of preparedness to deal with COVID-19-positive or suspected patients was filled by the participating doctors, where a score of 10 represented “fully prepared” while 0 represented “not prepared at all”. For ethical considerations, names of doctors and institutional information were not collected and data was used solely for statistical analysis.

### 2.3. Data Collection

The study design and its questionnaire were approved by the institutional review board (IRB) committee at the University of Jordan (reference number: 10/2020/7409). Data was collected in the period between March 23 to March 27. Based on phone and email communications with the designated institutes, the team of the study was able to identify 571 doctors as frontline doctors. All 571 doctors were approached by phone and email; 308 doctors filled the questionnaire and the consent form attached, marking a response rate of 53.9%.

### 2.4. Data Analysis

The authors analyzed the data using Statistical Package for Social Science program (SPSS) version 23.0 (SPSS Inc., Chicago, Ill., USA). We used Pearson’s Chi-squared (χ2) test for categorical variables. Independent t-test was used to investigate for significant associations between self-reported preparedness score, knowledge score, and adherence score with gender, presence of institutional protocol for dealing with COVID-19 patients, availability of PPE, psychological interactions, institutional support, and previously dealing with COVID-19-positive or suspected patients. One-way ANOVA (Analysis of Variance) followed by post hoc analysis of the least significant difference was used to compare between different workplaces, departments, and job descriptions in the preparedness score. Moreover, linear regression analysis and Pearson’s correlation coefficient (Pearson’s r) were used to explore the association between self-reported preparedness score and age, knowledge score, and adherence score. The statistical significance level was considered as a *p*-value less than 0.05. For questionnaire validation, the questionnaire was reviewed by seven anesthesiologists and by one doctor from the department of infectious diseases and was modified based on their comments. Calculated Cronbach’s alpha value was 0.81, marking a good level of internal consistency [13].

## 3. Results

Overall, 308 doctors with a mean age of 30.3 ± 5.8 were enrolled in the study, of which 195 (63.3%) were males and 113 (36.7%) were females. Most of the included frontline doctors were resident doctors (n = 174; 56.5%), followed by general practitioners (n = 73; 23.7%) and specialists (n = 61; 19.8%). Eighty-nine doctors (28.9%) were from emergency medicine and accidents departments, 87 (28.2%) were from anesthesia and intensive care departments, 74 (24%) were from internal medicine departments, 37 (12%) were from family medicine departments, and 21 (6.8%) were from ENT departments. Seventy-seven doctors (25%) previously took care of a positive or suspected COVID-19 patient, and their most trusted source of information was articles published in scientific journals (n = 267; 86.7%). The knowledge and adherence scores of these doctors were 8 ± 1.3 and 8.4 ± 1.5, respectively (Table 1). Moreover, the doctors’ psychological interactions and institutional support are explored in Table 2. 

The self-reported score of preparedness to deal with COVID-19-positive or suspected patients was 4.9 ± 2.4 (Table 2). Upon analyzing the effect of demographic factors, knowledge score, and adherence score on the self-reported preparedness score of frontline doctors, we found that males had higher preparedness scores (5.2 ± 2.4) when compared to females (4.5 ± 2.4; *p* = 0.019). Moreover, those who have an institutional protocol for dealing with COVID-19 suspected and confirmed cases at their institution scored 5.5 ± 2.3 (*p* < 0.001), and those who have sustained availability of PPE had significantly higher preparedness scores (6.2 ± 2.1; *p* < 0.001). Additionally, preparedness scores of doctors who previously took care of positive or suspected COVID-19 patients followed the same trend (*p* = 0.021), with a mean score of 5.5 ± 2.3 (Table 3). Doctors who were concerned about dealing with COVID-19 patients had higher knowledge scores (8.2 ± 1.3; *p* = 0.004). Likewise, those who feel anxious regarding the possibility of the spread of COVID-19 and the increase in number of positive patients had also higher scores (8.1 ± 1.3; *p* = 0.033) (Table 4).

Remarkably, those who have an institutional protocol for dealing with COVID-19 suspected and confirmed cases at their institution had a significantly higher percent of satisfaction with the infection control policy at their institutions, with feeling safe at their work, with feeling safe for their colleagues at work, and with feeling that current infection control practices at their institution will decrease the risk for them and their colleagues to contract COVID-19 (*p* < 0.001) (Table 5). Doctors who reported full availability of PPE followed the same positive trend for those four factors (*p* < 0.001) (Table 6). On the other hand, doctors who do not have all PPEs available at their institutions were significantly more concerned about dealing with COVID-19-positive or suspected patients (n = 177; 70.5%) when compared to those who always have PPEs available (n = 32; 56.1%; *p* = 0.036). As mentioned earlier, the full availability of PPEs was associated with higher self-reported preparedness scores (6.2 ± 2.1; *p* < 0.001). 

## 4. Discussion

Frontline doctors’ preparedness relies on two main pillars: self-preparedness and institutional preparedness. Self-preparedness depends on the amount of knowledge about the virus and the safe approach to patients and the amount of adherence to safety measures. Institutional preparedness is reflected by making safety measures available for doctors and by providing clear protocols to deal with COVID-19 patients. Psychological health and impacts on doctors during outbreaks should be targeted as an important factor of preparedness. The study evaluated self, institutional, and psychological preparedness of frontline doctors.

Studies conducted on healthcare system preparedness to outbreaks have long encouraged policy makers to modify policies based on findings and recommendations. In Jordan and many other countries, disease control and prevention committees are in charge of responding to public crises caused by viruses like COVID-19 [14]. Along with the ministry of health, they are also responsible for the fortification of capabilities of healthcare workers. Committees employ data of relevant studies to formulate these recommendations. Recently, with the COVID-19 pandemic, many studies are conducted worldwide to evaluate the readiness and the action measures applied to deal with pandemics [15]. Previous studies on the awareness of COVID-19 in healthcare workers worldwide showed that a significant proportion had poor knowledge about the virus yet positive perceptions about its control [16]. 

Our study included 308 doctors from all healthcare sectors across the country. Correlations regarding the job description were not valid as we assume all doctors who might be in first contact with a COVID-19 patient should have the same degree of preparedness. Institutional differences were omitted as many doctors might change their workplace according to needs during pandemics.

Scientific journals are believed to be the most trustful source of scientific information across all scientific communities; 59.7% of Jordanian doctors believe that officials are a trustful source, which reflects an adequate mutual trust between officials and frontline doctors. Only 16.9% of doctors identified social media as a safe source of information, which reflects the spread of fake science and news across Jordanian social media. Considering medical news, studies showed that at least 40% of information shared on social media is fake, of which 20% is “dangerously” fake [17].

For knowledge, a score of five fundamental questions was built to evaluate the knowledge needed to approach COVID-19 patients safely. Frontline doctors achieved satisfactory numbers with a mean of 8 ± 1.3. The biggest defect in terms of choices was that 58.1% of doctors do not consider the fecal-oral route as a possible route of disease transmission [18]. The biggest defect in terms of questions was in the “measures related to CPR” question, which can be attributed to the involvement of only specific specialties in the CPR team. Nevertheless, frontline doctors should acquire knowledge about all emergency situations [19]. As COVID-19 is an emerging disease, researches continue to fortify knowledge about it and institutions are recommended to update their healthcare workers on any new information [20].

Regarding adherence to safety measures, a score of three fundamental questions was built based on measures practiced before, during, and after work. The mean score of doctors was 8.4 ± 1.5, which is satisfactory; 65.6% of doctors are not adherent to any sport activity. There is a tremendous evidence in the literature linking healthy lifestyles to boosted immunity [21], which should be encouraged in healthcare communities.

As the number of positive cases is rising worldwide, the burden on healthcare systems is enlarging, which will increase the demand on medical supplies. Many facilities worldwide are suffering shortage in equipment supplies [22]. Only 18.5% of frontline doctors in Jordan reported that all protective measures are available, which reflects that the rest are at very high risk of catching the disease if PPE measures are not fully met [12]. Most shortage was in protective facemasks (66.2%). Alternative methods shared throughout social media do not meet the proper standards and are not of proven safety [23]. Facemasks are frequently reported to be the most important measure in PPE for healthcare workers [24,25].

The aim of the study was to evaluate frontline doctors’ preparedness. On a score out of 10, doctors’ self-reported preparedness mean was 4.9 ± 2.4. As the mean is unpleasantly low, correlations to understand the reason were established. Male doctors felt more prepared, and this could be regarded to female doctors worrying about being at childbearing age, having more family concerns or more anxiety thoughts, which might affect females more than males naturally [26]. Differences in relation to job description, specialty department, healthcare sector, and knowledge and adherence scores were not significant. Doctors who reported the availability of clear institutional protocols to approach COVID-19 patients and the availability of all PPE measures had the highest preparedness scores. There are many potential benefits of having clear institutional guidelines for doctors, which include improvement of the quality of clinical decisions, reduction of uncertainty in approaching patients, avoidance of outdated practices, and reassurance of practitioners’ treatment policies [27]. Availability of equipment is an essential factor in proper application of protocols and thus strongly affects preparedness [22]. Doctors who previously dealt with positive patients felt much more prepared than other doctors, which reflects the important role experience can play.

Outbreaks carry many psychological impacts on healthcare workers. These impacts can influence the quality of the healthcare provided. Doctors experiencing anxiety and distress might develop unfavorable mental health outcomes that might affect their preparedness to provide proper care [28]. Our study showed worrying results regarding the psychological health of Jordan frontline doctors. Only 28.2% of doctors are satisfied with the infection control policy at their institution, and only 19.8% feel safe at their workplace. More than 90% of doctors are concerned about the probability of transmitting the disease to their noninfected patients or their families. Considering previous figures, it would be expected that 67.9% of sampled doctors are concerned about dealing with COVID-19 patients.

Knowledge can significantly affect psychological impact. In our case, doctors with higher knowledge scores were more concerned about dealing with COVID-19 patients and more anxious regarding the increase of positive cases. This can be attributed to the proper understanding of the genuineness of the virus and to the lack of effective treatment policies till present time [29]. This also goes in line with findings in other studies that prove that poor knowledge is associated with less concerns [16]. The availability of clear protocols and full PPEs significantly improved figures of psychological impacts in terms of feeling safe at work and satisfaction about institutional plans; this emphasizes the importance of adopting international and local protocols at the institutional level and ensuring their proper application to avoid endangering the doctors [22]. Doctors without full PPEs were significantly more concerned about dealing with COVID-19 patients, which further expands the effect of shortage of PPE to fear and anxiety.

With progressing shortage in PPE, doctors may battle the indicated situations to adopt full PPEs; however, this should not be at the expense of doctors’ safety [30]. Different healthcare sectors have different capabilities to provide doctors’ needs, but in the case of an outbreak, unified protocols adopted by the highest healthcare authorities should be obligatory and frequently monitored for efficiency all over the country. As many countries are employing all healthcare sectors in the care of COVID-19 patients, preparedness of doctors in these sectors should be at the head of all priorities.

In 2009, avian influenza and pandemic influenza took place in Jordan and other countries around the world. According to the WHO (World Health Organization) Regional Office for the Eastern Mediterranean report, Jordan’s response to the pandemic had strong national communication and surveillance strategies and its national influenza center has become a regional reference laboratory in the region [31]. Nevertheless, many gaps have been identified in the policies and practices of the Ministry of Health in regard to effective risk communication with the public and healthcare workers during outbreaks [31]. In the new COVID-19 outbreak, major governmental efforts relying on WHO guidelines were made to fill these gaps, some of which had clearly contributed to the flattening of the epidemic curve, but the deficiencies recognized by this study may lead to a loophole that can overthrow these efforts.

The main limitation of this study is that the number of physicians interacting with COVID-19 patients is dynamically changing, which will have a continuous impact on their knowledge, their adherence to infection control policy, and their social and institutional support. However, the study illustrated the need for plans to take place for the current pandemic, and for actions that need to take place to prepare for future pandemics.

## 5. Conclusions

The study revealed multiple challenges and difficulties that can significantly affect frontline doctors’ preparedness. Policy makers in Jordan are urged to take these findings into consideration and to act abruptly. 

## Figures and Tables

**Table 1:** Demographics, awareness about COVID-19 (Coronavirus Disease of 2019) infection control practices, and adherence of frontline Jordanian doctors to common safety measures during COVID-19 pandemic.

Characteristic		Values
**Age (mean ± SD)**		30.3 ± 5.8
**Gender**	Female	113 (36.7)
	Male	195 (63.3)
**Job description**	General practitioner	73 (23.7)
	Resident doctor	174 (56.5)
	Specialist	61 (19.8)
**Current workplace**	Government hospital/healthcare facility	74 (24)
	Military hospital/healthcare facility	56 (18.2)
	Private hospital/healthcare facility	87 (28.2)
	University hospital	91 (29.5)
**Department**	Anesthesia and intensive care	87 (28.2)
	Emergency medicine and accidents	89 (28.9)
	ENT	21 (6.8)
	Family medicine	37 (12)
	Internal medicine	74 (24)
**Safe resource for information about COVID-19**	Local news	66 (21.4)
	Social media	52 (16.9)
	Officials	184 (59.7)
	Scientific journal articles	267 (86.7)
**Knowledge about COVID-19 infection control practices**		
**Routes believed to be potential sources for COVID-19 transmission**	Air	255 (82.8)
	Skin	200 (64.9)
	Fecal-oral	129 (41.9)
	Eyes	226 (73.4)
**Approaches thought to help prevent transmission of COVID-19**	Hand hygiene	302 (98.1)
	Covering nose and mouth while coughing	294 (95.5)
	Avoiding sick contacts	298 (96.8)
	Avoiding crowded places	296 (96.1)
**PPE for approaching a patient with suspected COVID-19 infection**	FFP3 mask	297 (96.4)
	Double gloves	271 (88)
	Gowns	279 (90.6)
	Visor (goggles)	258 (83.8)
**Considerations in CPR for patients diagnosed with or suspected to have COVID-19**	Avoid rescue breaths	247 (80.2)
	Avoid listening or feeling for breaths	178 (57.8)
	Tighten mask seal if intubation fails	209 (67.9)
	Let the most experienced person seek vascular access	217 (70.5)
**Considerations after dealing with suspected patient**	Remove all your PPE	266 (86.4)
	Get rid of disposable equipment	284 (92.2)
	Clean other equipment with chlor clean wipes	226 (73.4)
	Rubbish should be double bagged	208 (67.5)
***Total score for knowledge about COVID-19 (out of 10)***		8 ± 1.3
**Adherence to safety measures**		
**Safety measures they practice before commencing work**	Remove watch and jewelries	284 (92.2)
	Remove nail polish/cut nails	236 (76.6)
	Carry your personal stuff in washable bags	246 (79.9)
	Clean your scrubs	271 (88)
**Safety measures that they practice during work**	Sanitize your phone, stethoscope, badge, bed, and room	267 (86.7)
	Hand washing/hygiene before and after dealing with every patient	302 (98.1)
	Avoid handshakes and high fives	289 (93.8)
	Wear appropriate PPE when indicated always	270 (87.7)
**Safety measures that they practice after finishing work**	Wash your bag, clothes, and lunch Tupperware and sanitize your stuff	283 (91.9)
	Leave shoes at work or outside home	282 (91.6)
	Shower immediately at home	272 (88.3)
	Do any sport activity	106 (34.4)
***Total score for Adherence to safety measures (out of 10)***		8.4 ± 1.5
**Have specific institutional protocol to approach COVID-19 patients**		173 (56.2)
**PPE not always available at their institution**	FFP3 mask	204 (66.2)
	Double gloves	52 (16.9)
	Gowns	94 (30.5)
	Visor (goggles)	203 (65.9)
	All PPE are always available	57 (18.5)
**Previously took care of a positive or suspected COVID-19 patient**		77 (25)

SD: standard deviation; ENT: ear, nose throat; COVID-19: Coronavirus disease 2019; PPE: personal protective equipment; FFP3 mask: Filtering Face Piece-3 mask; CPR: cardiopulmonary resuscitation. Values are represented as number (percent) and mean ± SD forms.

**Table 2:** Psychological interactions, institutional support, and self-reported preparedness for dealing with COVID-19 patients among frontline doctors.

Characteristic	Values
**Satisfied with the infection control policy at their institution**	87 (28.2)
**Feel safe at work with the current safety precautions**	61 (19.8)
**Feel safe about their colleagues with the current safety precautions**	71 (23.1)
**Feel that current infection control practice at their institution will decrease the risk for them and their colleagues to contract COVID-19**	106 (34.4)
**Concerned of dealing with COVID-19 patients**	209 (67.9)
**Feel anxious regarding the possibility of the spread of COVID-19 and increase in the number of positive patients**	280 (90.9)
**Afraid of the transmission of COVID-19 to their patients who are not diagnosed/suspected to have COVID-19**	288 (93.5)
**Afraid of the transmission of COVID-19 to their families**	297 (96.4)
**Self-reported score of preparedness to deal with COVID-19-positive/suspected patients (mean ± SD)**	4.9 ± 2.4

SD: standard deviation; COVID-19: Coronavirus disease 2019. Values are represented as number (percent) and mean ± SD forms.

**Table 3:** An analysis on the effect of demographic factors, knowledge score, and adherence score on the self-reported preparedness score of frontline doctors.

Characteristic		Number (Percent)	Preparedness Score	*p*-Value
**Age**		30.3 ± 5.8	r = −0.015	0.793
**Gender**	Male	195 (63.3)	5.2 ± 2.4	0.019
	Female	113 (36.7)	4.5 ± 2.4	
**Job description**	General practitioner	73 (23.7)	5.4 ± 2.3	0.072
	Resident doctor	174 (56.5)	4.7 ± 2.4	
	Specialist	61 (19.8)	5 ± 2.5	
**Current workplace**	Government hospital/healthcare facility	74 (24)	4.8 ± 2.5	0.639
	Military hospital/healthcare facility	56 (18.2)	5.2 ± 2.7	
	Private hospital/healthcare facility	87 (28.2)	5.0 ± 2.4	
	University hospital	91 (29.5)	4.8 ± 2.2	
**Department**	Anesthesia and intensive care	87 (28.2)	4.4 ± 2.5	0.092
	Emergency department	89 (28.9)	4.9 ± 2.3	
	ENT	21 (6.8)	5.1 ± 1.9	
	Family medicine	37 (12)	5.6 ± 2.4	
	Internal medicine	74 (24)	5.1 ± 2.5	
**COVID-19 patient care protocol available**	Yes	173 (56.2)	5.5 ± 2.3	<0.001
	No	135 (43.8)	4.1 ± 2.4	
**All PPEs are always available at their institution**	Yes	57 (18.5)	6.2 ± 2.1	<0.001
	No	251 (81.5)	4.6 ± 2.4	
**Previously took care of a positive or suspected COVID-19 patient**	Yes	77 (25)	5.5 ± 2.3	0.021
	No	231 (75)	4.7 ± 2.4	
**Knowledge score**		8 ± 1.3	r = −0.04	0.482
**Adherence to safety measures score**		8.4 ± 1.5	r = −0.008	0.889

ENT: ear, nose throat; COVID-19: Coronavirus disease 2019; r: Pearson correlation coefficient (Pearson’s r). Knowledge score: Total score for the section investigating knowledge about COVID-19 (out of 10) (Table 1).

**Table 4:** The influence of knowledge about COVID-19 infection control practices on the psychological interactions and preparedness of frontline Jordanian doctors.

Characteristic		Number (Percent)	Knowledge Score	*p*-Value
**Satisfied with the infection control policy at their institution**	Yes	87 (28.2)	7.8 ± 1.5	0.118
	No	221 (71.8)	8.1 ± 1.2	
**Feel safe at work with the current safety precautions**	Yes	61 (19.8)	8 ± 1.3	0.94
	No	247 (80.2)	8 ± 1.3	
**Feel safe about their colleagues with the current safety precautions**	Yes	71 (23.1)	7.9 ± 1.4	0.51
	No	237 (76.9)	8 ± 1.3	
**Feel that current infection control practice at their institution will decrease the risk for them and their colleagues to contract COVID-19**	Yes	106 (34.4)	7.9 ±1.3	0.215
	No	202 (65.6)	8.1 ± 1.3	
**Concerned of dealing with COVID-19 patients**	Yes	209 (67.9)	8.2 ± 1.3	0.004
	No	99 (32.1)	7.7 ± 1.3	
**Feel anxious regarding the possibility of the spread of COVID-19 and increase in the number of positive patients**	Yes	280 (90.9)	8.1 ± 1.3	0.033
	No	28 (9.1)	7.5 ± 1.2	
**Afraid of the transmission of COVID-19 to their patients who are not diagnosed/suspected to have COVID-19**	Yes	288 (93.5)	8 ± 1.3	0.493
	No	20 (6.5)	7.8 ± 1.3	
**Afraid of the transmission of COVID-19 to their families**	Yes	297 (96.4)	8 ± 1.3	0.324
	No	11 (3.6)	7.6 ± 1.3	

PPE: personal protective equipment; COVID-19: Coronavirus disease 2019. Knowledge score: Total score for the section investigating knowledge about COVID-19 (out of 10) (Table 1).

**Table 5:** The influence of the presence of a protocol for dealing with COVID-19 patients on the psychological interactions and preparedness of frontline Jordanian doctors.

Characteristics	Protocol Availability		Total	*p*-Value
	Available (n = 173)	Not Available (n = 135)		
**Satisfied with the infection control policy at their institution**	66 (38.2)	21 (15.6)	87 (28.2)	<0.001
**Feel safe at work with the current safety precautions**	50 (28.9)	11 (8.1)	61 (19.8)	<0.001
**Feel safe about their colleagues with the current safety precautions**	54 (31.2)	17 (12.6)	71 (23.1)	<0.001
**Feel that current infection control practice at their institution will decrease the risk for them and their colleagues to contract COVID-19**	80 (46.2)	26 (19.3)	106 (34.4)	<0.001
**Concerned of dealing with COVID-19 patients**	114 (65.9)	95 (70.4)	209 (67.9)	0.404
**Feel anxious regarding the possibility of the spread of COVID-19 and increase in the number of positive patients**	154 (89)	126 (93.3)	280 (90.9)	0.191
**Afraid of the transmission of COVID-19 to their patients who are not diagnosed/suspected to have COVID-19**	159 (91.9)	129 (95.6)	288 (93.5)	0.197
**Afraid of the transmission of COVID-19 to their families**	164 (94.8)	133 (98.5)	297 (96.4)	0.081

SD: standard deviation; COVID-19: Coronavirus disease 2019.

**Table 6:** The influence of the availability of PPE on the psychological interactions and preparedness of frontline Jordanian doctors.

Characteristics	Availability of PPE		Total	*p*-Value
	All Are Always Available n = 57	Not Always Available n = 251		
**Satisfied with the infection control policy at their institution**	40 (70.2)	47 (18.7)	87 (28.2)	<0.001
**Feel safe at work with the current safety precautions**	29 (50.9)	32 (12.7)	61 (19.8)	<0.001
**Feel safe about their colleagues with the current safety precautions**	29 (50.9)	42 (16.7)	71 (23.1)	<0.001
**Feel that current infection control practice at their institution will decrease the risk for them and their colleagues to contract COVID-19**	39 (68.4)	67 (26.7)	106 (34.4)	<0.001
**Concerned about dealing with COVID-19 patients**	32 (56.1)	177 (70.5)	209 (67.9)	0.036
**Feel anxious regarding the possibility of the spread of COVID-19 and increase in the number of positive patients**	51 (89.5)	229 (91.2)	280 (90.9)	0.676
**Afraid of the transmission of COVID-19 to their patients who are not diagnosed/suspected to have COVID-19**	50 (87.7)	238 (94.8)	288 (93.5)	0.05
**Afraid of the transmission of COVID-19 to their families**	55 (96.5)	242 (96.4)	297 (96.4)	0.977

PPE: personal protective equipment; COVID-19: Coronavirus disease 2019.
